# Copper-Catalyzed *N*-Arylation of Amides Using (S)-*N*-Methylpyrrolidine-2-carboxylate as the Ligand

**DOI:** 10.3390/molecules15031154

**Published:** 2010-03-02

**Authors:** Chaoyu Wang, Lijuan Liu, Wei Wang, Dong-Sheng Ma, Hua Zhang

**Affiliations:** 1 College of Chemical Engineering and Material, Heilongjiang University, Haerbin 150080, China; E-Mails: wgyxhz@126.com (C.W.); liulijuan1972@yahoo.com.cn (L.L.); dms_70@163.com (D.M.); amewangwei@yahoo.cn (W.W.); 2 Mudanjiang Normal University, Mudanjiang 157012, China

**Keywords:** copper, (S)-*N*-methylpyrrolidine-2-carboxylate, arylations, amides

## Abstract

(S)-*N*-methylpyrrolidine-2-carboxylate, a derivative of natural L-proline, was found to be an efficient ligand for the copper-catalyzed Goldberg-type *N*-arylation of amides with aryl halides under mild conditions. A variety of *N*-arylamides were synthesized in good to high yields.

## 1. Introduction

**Figure 1:**
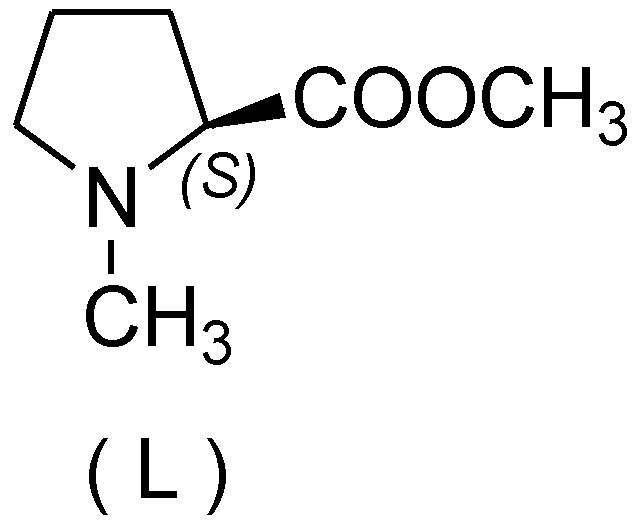
(*S*)-*N*-methylpyrrolidine-2-carboxylate (ligand).

*N*-Arylamides are valuable compounds widely employed in the fields of organic synthesis, pharmaceutical chemistry, or biology [1,2,3]. One of the most common synthetic protocols for their preparation is the copper-catalyzed Ullmann reaction [4] and the related Goldberg reaction (copper-catalyzed *N*-arylation of amides) [5]. However, classic Ullmann reactions are usually conducted under harsh conditions, and therefore their applications would be restricted. In recent years, some efficient ligands [6,7,8,9] have been disclosed for copper-catalyzed *N*-arylation under mild conditions including diamines [10,11,12], diimines [13], amino acids [14,15], β-keto esters [16], and diols [17]. Some ligand-free Ullmann-type coupling reactions have also been reported [18,19,20,21,22]. However, it is well accepted that some reactions require 10 mol% of copper as a catalyst and long reaction times for the reaction to proceed accordingly. We now report the use of (*S*)-*N*-methylpyrrolidine-2-carboxylate (Figure 1), a derivative of natural L-proline, as the ligand of 5 mol % copper catalyst in the *N*-arylation of amides. Satisfactory results were obtained under mild conditions and short reaction time.

**Table 1:**
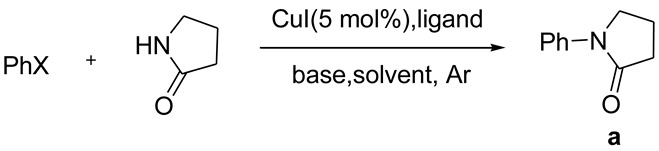
Optimization of reaction conditions for *N*-phenylation of 2-pyrrolidone.

Entry	Substrate	Ligand	Base	Solvent	Temperature (°C)	Time (h)	Yield (%)
1	PhI	L	KO*t*-Bu	DMF	110	5	10
2	PhI	L	KOH	DMF	110	5	13
3	PhI	L	Cs_2_CO_3_	DMF	110	5	60
4	PhI	L	K_2_CO_3_	DMF	110	5	61
5	PhI	L	Na_2_CO_3_	DMF	110	5	63
6	PhI	L	Li_2_CO_3_	DMF	110	5	66
7	PhI	L	K_3_PO_4_	DMF	110	5	76
8	PhI	L	K_3_PO_4_	DMF	110	7	76
9	PhI	L	K_3_PO_4_	DMF	120	5	75
10	PhI	L	K_3_PO_4_	DMF	90	5	62
11	PhI	-	K_3_PO_4_	DMF	110	5	15
12	PhI	L	K_3_PO_4_	DMSO	110	5	90
13	PhI	L	K_3_PO_4_	DMSO	110	7	90
14	PhBr	L	K_3_PO_4_	DMSO	110	5	11
15	PhCl	L	K_3_PO_4_	DMSO	110	5	3

## 2. Results and Discussion

The ligand (*S*)-*N*-methylpyrrolidine-2-carboxylate was synthesized according to the reported procedure [23]. Initially, we tried to seek the optimal reaction conditions for copper-catalyzed *N*-arylation of amides. Aryl halides and 2-pyrrolidone were chosen as the model substrates with 5 mol % of CuI as a catalyst under an argon atmosphere at 110 °C (Table 1). In the presence of (S)-*N*-methylpyrrolidine 2-carboxylate as a ligand and DMF as a solvent, K_3_PO_4_ was found to be the most appropriate base, 110 °C to be the most appropriate reaction temperature, and 5 hours to be the most appropriate reaction time (entries 1–10). Without using the ligand, the yield was greatly decreased (entry 11). The effect of various aryl halides and solvents was investigated (entries 7,12,14,15). The use of aryl iodides as substrate and DMSO as a solvent led to a best result for this reaction (entry 12). Based on those results, the effect of Goldberg-type *N*-arylations of various amides with various aryl iodides was investigated (entries 1–11, Table 2). Using 4-substituted aryl iodides led to good yields of the desired products (entries 1–4). Moreover, 2-iodotoluene and 1-iodonaphthalene also led to good yields (entries 5,6). 

**Table 2:**
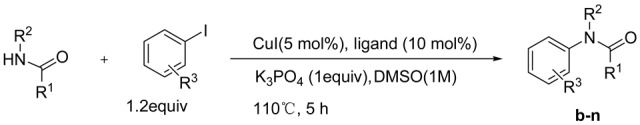
Copper-catalyzed *N*-arylations of amides with aryl iodides.

Entry	Aryl iodide	Amide	Products	Yield (%) ^a^
1	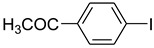	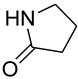	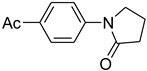 **b**	90
2	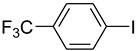	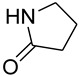	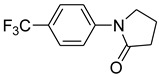 **c**	89
3	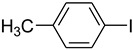	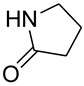	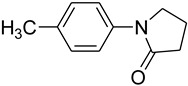 **d**	93
4	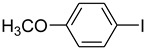	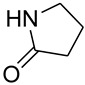	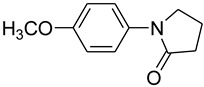 **e**	88
5	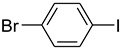	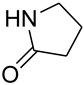	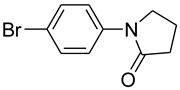 **f**	89
6	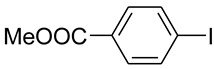	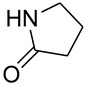	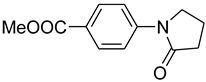 **g**	85
5	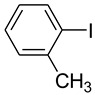	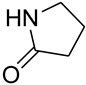	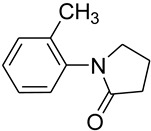 **h**	86
6	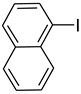	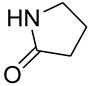	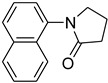 **i**	93
7	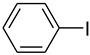	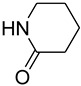	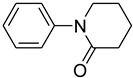 **j**	81
8	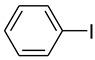	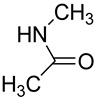	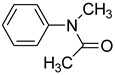 **k**	79
9	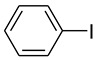	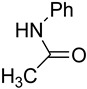	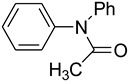 **l**	75
10	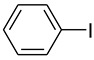	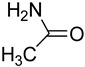	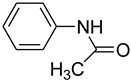 **m**	76 (10 ^b^)
11	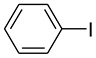	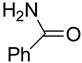	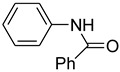 **n**	80 (11 ^b^)

Note: ^a^ Isolated yield, ^b^ bisphenylated yield.

The *N*-phenylation of various amides gave the corresponding products with moderate to good yields (entries 7–11). In the reaction of acetamide, bisphenylation product was obtained accompanying the corresponding product (entries 10–11, Table 2). 

## 3. Experimental

### 3.1. General

All starting compounds were used as received from commercial sources without further purification. Petroleum ether (PE) used had the boiling range 60–90 °C. Melting points were determined on a XRC-1 micromelting point apparatus and are uncorrected. Column chromatography was carried out on silica gel (200–300 mesh, Qingdao Haiyang Chemical Co., Ltd.). NMR spectra were recorded at 300 MHz (^1^H) and 75 MHz (^13^C) in CDCl_3_ on a Bruker Avance 300 spectrometer with TMS as internal standard. MS spectra measurements were carried out on a Finnigan LCQDECA mass spectrometer (ESI-MS) and a BioTOF-Q mass spectrometer (HR-ESI-MS).

### 3.2. General procedure for copper-catalyzed N-arylations of amides with aryl iodides

Under an atmosphere of Ar, aryl iodide (12 mmol) was added to the mixture of amide (10 mmol), K_3_PO_4_ (10 mmol), ligand (1 mmol), and CuI (0.5 mmol) in DMSO (10 mL) at r.t. The mixture was stirred at 110 °C. After 5 h, the mixture was cooled to r.t. diluted with EtOAc, and filtered through Celite, eluting with additional EtOAc. The filtrate was concentrated under reduced pressure, and the resulting residue was purified by silica gel chromatography using a mixture of hexane and EtOAc as eluent. All prepared compounds **a-n** are known and identified by ^1^H- and ^13^C-NMR and MS.

*1-Phenylpyrrolidin-2-one* (**a**): mp 68–69 °C. ^1^H-NMR: δ = 2.12 (m, 2H), 2.45 (t, *J* = 7.5 Hz, 2H), 3.31 (t, *J* = 7.1 Hz, 2H), 7.00 (d, *J* = 8.0 Hz, 2H), 7.21 (m, 1H), 7.46 (m, 2H). ^13^C NMR: δ = 18.1, 32.7, 49.1, 122.1, 125.3, 131.2, 142.5, 175.3. MS (EI): *m/z* = 161 (100) [M^+^]. HRMS: *m/z* calcd for C_10_H_11_NO: 161.2005; found: 161.2013.

*1-(4-Acetylphenyl)pyrrolidin-2-one* (**b**): mp 91–93 °C. ^1^H-NMR: δ = 2.16 (m, 2H), 2.38 (t, *J* = 6.5 Hz, 2H), 2.56 (s, 3H), 3.45 (t, *J* = 6.8 Hz, 2H), 7.62 (d, *J* = 7.6 Hz, 2H), 8.10 (d, *J* = 8.7 Hz, 2H). ^13^C- NMR: δ = 17.1, 30.3, 32.5, 48.6, 122.3, 129.8, 133.1, 146.6, 175.2, 197.6. MS (EI): *m/z* = 203 (100) [M^+^]. HRMS: *m/z* calcd for C_12_H_13_NO_2_: 203.2372; found: 203.2365.

*1-(4-Trifluoromethylphenyl)pyrrolidin-2-one* (**c**): mp 86–88 °C. ^1^H-NMR: δ = 2.15 (m, 2H), 2.58 (t, *J* = 7.3 Hz, 2H), 3.32 (t, *J* = 7.6 Hz, 2H), 7.00 (d, *J* = 7.2 Hz, 2H), 7.65 (d, *J* = 8.6 Hz, 2H). ^13^C-NMR: δ = 18.5, 33.6, 48.9, 122.1, 123.5, 125.0, 127.6, 147.5, 175.8. MS (EI): *m/z* = 229 (100) [M^+^]. HRMS: *m/z* calcd for C_1__1_H_1__0_F_3_NO: 229.1984; found: 229.1976.

*1-(4-Methylphenyl)pyrrolidin-2-one* (**d**): mp 73.5–75.1 °C. ^1^H-NMR: δ = 2.13 (m, 2H), 2.38 (s, 3H), 2.56 (t, *J* = 7.0 Hz, 2H), 3.21 (t, *J* = 6.3 Hz, 2H), 6.89 (d, *J* = 7.5 Hz, 2H), 7.12 (d, *J* = 8.5 Hz, 2H). ^13^C-NMR: δ = 17.6, 24.9, 33.1, 49.2, 122.1, 130.5, 134.7, 139.2, 175.8. MS (EI): *m/z* = 175 (100) [M^+^]. HRMS: *m/z* calcd for C_11_H_13_NO: 175.2271; found: 175.2262.

*1-(4-Methoxyphenyl)pyrrolidin-2-one* (**e**): mp 89.5–91.0 °C. ^1^H-NMR: δ = 2.15 (m, 2H), 2.55 (t, *J* = 7.6 Hz, 2H), 3.23 (t, *J* = 7.3 Hz, 2H), 3.71 (s, 3H), 3.806.80 (d, *J* = 7.6 Hz, 2H), 7.05 (d, *J* = 8.2 Hz, 2H). ^13^C-NMR: δ = 17.5, 32.6, 48.7, 56.2, 114.8, 122.1, 135.6, 156.9, 175.2. MS (EI): *m/z* = 191 (100) [M^+^]. HRMS: *m/z* calcd for C_1__1_H_13_NO_2_: 191.2265; found: 191.2261.

*1-(4-Bromophenyl)pyrrolidin-2-one* (**f**): mp 99.5–101.0 °C. ^1^H-NMR: δ = 2.20 (m, 2H), 2.50 (t, *J* = 7.2 Hz, 2H), 3.34 (t, *J* = 7.4 Hz, 2H), 6.83 (d, *J* = 7.5 Hz, 2H), 7.41 (d, *J* = 8.1 Hz, 2H). ^13^C-NMR: δ = 17.6, 33.2, 48.0, 118.1, 124.1, 132.4, 142.9, 175.0. MS (EI): *m/z* = 239 (100) [M^+^]. HRMS: *m/z* calcd for C_1__0_H_1__0_BrNO: 238.9912; found: 238, 9908.

*Methyl 4-(2-oxopyrrolidin-1-yl)benzoate* (**g**): mp 80.5–82.0 °C. ^1^H-NMR: δ = 2.21 (m, 2H), 2.61 (t, *J* = 7.0 Hz, 2H), 3.38 (t, *J* = 7.5 Hz, 2H), 3.91 (s, 3H), 6.83 (d, *J* = 7.6 Hz, 2H), 7.76 (d, *J* = 8.0 Hz, 2H). ^13^C-NMR: δ = 17.0, 33.2, 49.2, 52.3, 122.8, 126.2, 131.1, 147.2, 167.2, 175.3. MS (EI): *m/z* = 219 (100) [M^+^]. HRMS: *m/z* calcd for C_12_H_13_NO_2_: 219.0915; found: 219.0906.

*1-(2-Methylphenyl)pyrrolidin-2-one* **(h):** mp 74–75.2 °C. ^1^H-NMR: δ = 2.12 (m, 2H), 2.31 (t, *J* = 7.6 Hz, 2H), 2.38 (s, 3H), 3.42 (t, *J* = 7.4 Hz, 2H), 6.90 (d, *J* = 7.2 Hz, 1H), 7.00-7.21 (m, 3H). ^13^C-NMR: δ =14.8, 17.3, 33.5, 48.0, 122.3, 124.5, 126.3, 131.7, 134.0, 139.7, 175.2. MS (EI): *m/z* = 175 (100) [M^+^]. HRMS: *m/z* calcd for C_11_H_13_NO: 175.2271; found: 175.2265.

*1-(Naphthalen-1-yl)pyrrolidin-2-one* (**i**): mp 87.5–89.0 °C. ^1^H-NMR: δ = 2.05 (m, 2H), 2.34 (t, *J* = 7.5 Hz, 2H), 2.71 (t, *J* = 7.6 Hz, 2H), 6.50 (d, *J* = 6.8 Hz, 1H), 7.10–7.66 (m, 6H). ^13^C-NMR: δ =17.6, 33.5, 49.6, 110.2, 119.3, 121.6, 124.0, 124.9, 126.1, 126.9, 129.0, 135.1, 143.2, 176.2. MS (EI): *m/z* = 211 (100) [M^+^]. HRMS: *m/z* calcd for C_14_H_13_NO: 211.2592; found: 211.2590.

*1-Phenylpiperidin-2-one* (**j**): mp 71.3–72.1 °C. ^1^H-NMR: δ = 1.47 (m, 2H), 1.72 (m, 2H), 2.67 (t, *J* = 7.6 Hz, 2H), 3.36 (t, *J* = 7.2 Hz, 2H), 7.12 (d, *J* = 7.4 Hz, 2H), 7.22–7.40 (m, 3H). ^13^C-NMR: δ = 23.1, 27.1, 34.2, 49.1, 122.3, 123.8, 129.6, 142.5, 170.2. MS (EI): *m/z* = 175 (100) [M^+^]. HRMS: *m/z* calcd for C_11_H_13_NO: 175.2271; found: 175.2272.

*N-Methyl-N-phenylacetamide* (**k**): mp 100–101 °C. ^1^H-NMR: δ = 2.36 (s, 3H), 2.83 (s, 3H) 7.11 (d, *J* = 7.3 Hz, 2H), 7.26–7.38 (m, 3H). ^13^C-NMR: δ =21.2, 33.0, 122.6, 124.8, 129.8, 142.5, 171.3. MS (EI): *m/z* = 149 (100) [M^+^]. HRMS: *m/z* calcd for C_9_H_11_NO: 149.1898; found: 149.1893.

*N, N-Diphenylacetamide* (**l**): mp 101–103 °C. ^1^H-NMR: δ = 2.43 (s, 3H), 7.01–7.32 (m, 6H) 7.69 (d, *J* = 7.2 Hz, 4H). ^13^C-NMR: δ =21.2, 118.8, 119.6, 130.1, 141.3, 173.4. MS (EI): *m/z* = 211 (100) [M^+^]. HRMS: *m/z* calcd for C_14_H_13_NO: 211.2592; found: 211.2597.

*N-Phenylacetamide* (**m**): mp 155–156 °C. ^1^H-NMR: δ = 2.33 (s, 3H), 7.11–7.32 (m, 3H) 7.66 (d, *J* = 7.4 Hz, 4H), 9.12 (s, 1H). ^13^C-NMR: δ =23.1, 122.3, 124.7, 130.2, 140.0, 168.2. MS (EI): *m/z* = 135 (100) [M^+^]. HRMS: *m/z* calcd for C_8_H_9_NO: 135.1632; found: 135.1621.

*N-Phenylbenzamide* (**n**): mp 163–164.5 °C. ^1^H-NMR: δ = 6.92–7.30 (m, 3H), 7.40–7.58 (m, 3H), 7.70 (d, *J* = 7.2 Hz, 2H), 7.91 (d, *J* = 7.4 Hz, 2H). 9.01 (s, 1H). ^13^C-NMR: δ = 120.5, 123.2, 127.5, 129.1, 132.8, 134.6, 135.2, 166.5. MS (EI): *m/z* = 197 (100) [M^+^]. HRMS: *m/z* calcd for C_13_H_11_NO: 197.08646; found: 197.0841.

## 4. Conclusions

In summary, we have developed a simple and highly efficient ligand, (*S*)-*N*-methylpyrrolidine-2-carboxylate, that can promote the copper-catalyzed Goldberg-type *N*-arylation of amides with aryl iodides. Further work is in progress to examine its catalytic activity in other copper-catalyzed organic transformations.
